# Quantifying the Performance of Professional Athletes Following Traumatic Tibial Fractures

**DOI:** 10.7759/cureus.59198

**Published:** 2024-04-28

**Authors:** Ankit Punreddy, Paul Guirguis, Mina Botros

**Affiliations:** 1 Plastic Surgery, University of Rochester School of Medicine and Dentistry, Rochester, USA; 2 Orthopaedics, University of Rochester School of Medicine and Dentistry, Rochester, USA; 3 Orthopaedic Surgery, University of Rochester Medical Center, Rochester, USA

**Keywords:** return to sport, athletic performance, professional athletes, sports injury, tibial fracture

## Abstract

Introduction: A tibial fracture is an uncommon, yet severe injury that occurs in professional athletes within all major sports leagues. These injuries are often debilitating for professional athletes and can require extensive rehabilitation prior to returning to play. The purpose of this study is to investigate the impact of a tibial fracture on athletic performance in all four major United States sports leagues.

Methods: A publicly available professional sports database, Pro Sports Transactions, was queried for tibial injuries from January 01, 2015, to May 31, 2023. The search included all four major U.S. sports leagues. To quantify and compare athletic ability before and after injury, performance-based statistics were collected from standardized player ratings in periods surrounding the date of injury. The percent change in player performance was measured and stratified. The Pearson correlation test was used to analyze player demographics.

Results: There were a total of 24 professional athletes who suffered 28 confirmed tibial fractures across all leagues. Upon return, there was a 14.7% decrease in overall player performance across all leagues. National Basketball Association, National Football League, and National Hockey League athletes had a decrease of 34.5%, 29.1%, and 14.2%, respectively, following their return to play. Major League Baseball players demonstrated an 8.1% increase in player performance following their recovery from tibial fracture.

Conclusion: Players who suffer tibial fractures often undergo immediate surgery and, in unfortunate cases, may require multiple subsequent procedures. Additionally, athletes spend several months recovering prior to their return. Upon return, athletes' performance may be decreased; however, further study is required to strengthen the association between player performance and tibial fracture recovery.

## Introduction

The management of sports-related injuries is a mainstay of orthopedics [1]. Whether professional or amateur, athletes invest tremendous effort and dedication to achieve peak performance levels [2]. However, athletic endeavors often require repetitive and dynamic skeletal stress, and athletes frequently encounter injuries that can significantly impact their performance [3-13]. One such injury that can have profound implications on an athlete's capabilities is a tibial fracture, a relatively uncommon but debilitating lower limb injury at any level of sports [4,10]. Athletes who suffer from tibial fractures may face prolonged periods of rehabilitation, and the potential to return to optimal performance levels is unpredictable [2-4,8,10,14]. The uncertainty surrounding the impact of tibial fractures on the performance and length of recovery can be distressing to athletes and their organizations [11].

Previous literature is limited in this population and lacks measurement of athletic performance following tibial fracture [11]. A 2021 study by Knapik et al. investigated National Football League (NFL) players who suffered from tibial fractures with an interest in time from injury to return, mechanism of injury, and timing of the injury in the context of the NFL season [15]. Another study by Robertson et al. investigated whether athletes who suffered tibial stress fractures were able to return to play at all [16]. However, there is no previous literature that measures the direct impact of tibial injury on athletic performance before and after injury, particularly across all major United States (US) sports leagues. We aim to address this gap in the literature by quantifying athletic performance pre- and post-injury, with consideration of type of sport, mechanism of injury, and recovery length.

By completing this investigation, we aim to provide insights to guide professional athletes, coaches, and medical providers in developing more effective injury prevention strategies, rehabilitation protocols, and personalized recovery plans. At the very least, compiled data may provide realistic expectations regarding recovery length and impact on performance to athletes and organizations in the uncertain and distressing period following a tibial fracture.

## Materials and methods

This study utilized data from a publicly accessible professional sports database, Pro Sports Transactions, to identify “tibia” and “shin” injuries occurring between January 1, 2015, and May 31, 2023 [17]. This database encompassed injuries from the four major US sports leagues, namely, the NFL, the National Basketball Association (NBA), Major League Baseball (MLB), and the National Hockey League (NHL). Once injuries were identified, information was gathered from various sources, including team injury reports, media coverage, and player records, to construct a comprehensive list for each athlete involved. All data retrieved from media outlets were cross-referenced with multiple sources. The compiled data included details such as demographics, injury mechanisms, injury dates, surgical procedures, and return-to-play dates.

To quantify the impact of tibial fracture on athletic ability, each athlete’s season statistics were collected one season before injury and the season of return. Performance for each season was based on average fantasy points per game over each season. Season fantasy data assign individual achievements, such as goals scored, to a weighted numerical point value detailed below (Appendix 1). These statistics were retrieved from an online archive, FantasyData.com [18]. All retrieved values were cross-referenced with another online archive, StatMuse.com, to ensure accuracy [19].

A comparative analysis was conducted to determine the extent of change in performance before and after injury occurrence. Notably, the season of injury was excluded from this analysis to ensure accurate measurements. The observed percentage change in player performance was then categorized based on stratification criteria. To assess potential relationships between demographic factors and injury outcomes, a Pearson correlation test was employed. This analysis allowed for the examination of any significant associations between demographic variables and the severity or recovery outcomes of tibia and shin injuries.

## Results

**Figure 1:**
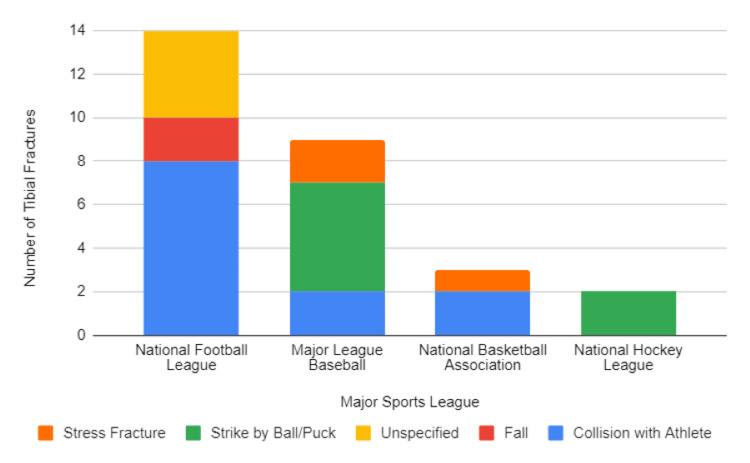
Number of tibial fractures in each US sports league from 01/01/2015 to 05/31/2023

From January 01, 2015, to May 31, 2023, a total of 24 professional athletes from four major sports leagues sustained 28 confirmed tibial fractures (Table 1). Among these injuries, the highest number of fractures occurred in the NFL (14 fractures), followed by MLB (nine fractures), NBA (three fractures), and NHL (two fractures), respectively. Surgical intervention was performed for 16 of the 28 injuries, all of which occurred within 24 hours of fracture. However, two out of 16 surgeries resulted in postoperative complications and required multiple additional surgical interventions (Figure 1).

**Table 1:** Recorded fractures with athlete demographics and details of injury MCL: medial collateral ligament

	League	Position	Height (in)	Weight (lb)	Age at injury	Days from injury to return	Mechanism of injury	Description of the fracture	Associated ipsilateral limb injuries	Surgery (Y/N)
Athlete 1	National Football League	Fullback	75	251	27	349	Collision with athlete	Clean break	-	Y
Athlete 2	National Football League	Running back	70	220	25	419	Unspecified	Open fracture	-	Y
Athlete 2	-	-	-	-	26	111	Unspecified	-	-	Y
Athlete 2	-	-	-	-	26	Did not return	Unspecified	-	-	Y
Athlete 3	National Football League	Wide receiver	70	182	24	260	Collision with athlete	-	Fibula fracture	Y
Athlete 4	National Football League	Defensive end	77	288	28	336	Fall	Tibial plateau fracture	-	Y
Athlete 5	National Football League	Cornerback	72	190	26	405	Collision with athlete	-	Fibula fracture	Y
Athlete 6	National Football League	Free safety	70	202	27	280	Collision with athlete	-	-	-
Athlete 6	-	-	-	-	29	Did not return	Fall	-	-	Y
Athlete 7	National Football League	Wide receiver	69	176	24	252	Collision with athlete	-	-	Y
Athlete 7	-	-	-	-	25	338	Unspecified	-	-	-
Athlete 8	National Football League	Quarterback	76	216	34	693	Collision with athlete	Compound fracture	Fibula fracture	Y, multiple
Athlete 9	National Football League	Center	77	306	25	409	Collision with athlete	-	Fibula fracture	Y, multiple
Athlete 10	National Football League	Cornerback	72	197	24	Did not return	Collision with athlete	-	Fibula fracture	Y
Athlete 11	Major League Baseball	Infielder	72	195	25	317	Strike by ball/puck	Hairline fracture	-	-
Athlete 12	Major League Baseball	Infielder	68	184	25	269	Strike by ball/puck	Stress fracture	-	Y
Athlete 13	Major League Baseball	Infielder	72	210	28	232	Collision with athlete	Displaced lateral tibial plateau fracture	MCL and lateral meniscus tear	Y
Athlete 14	Major League Baseball	Outfielder	74	210	33	160	Strike by ball/puck	-	-	-
Athlete 15	Major League Baseball	Infielder	70	210	25	724	Collision with athlete	-	-	-
Athlete 16	Major League Baseball	Pitcher	73	190	27	41	Stress fracture	Stress fracture	Fibula fracture	-
Athlete 17	Major League Baseball	Infielder	71	185	30	86	Strike by ball/puck	-	-	-
Athlete 18	Major League Baseball	Pitcher	74	235	28	44	Stress fracture	Stress fracture	-	-
Athlete 19	Major League Baseball	Infielder	72	160	27	Did not return	Strike by ball/puck	-	-	-
Athlete 20	National Hockey League	Center	73	185	29	127	Strike by ball/puck	Complete break	-	-
Athlete 21	National Hockey League	Right winger	70	185	24	86	Strike by ball/puck	Hairline fracture	-	-
Athlete 22	National Basketball Association	Center	84	270	25	355	Stress fracture	Stress fracture	-	Y
Athlete 23	National Basketball Association	Center	84	260	32	225	Collision with athlete	-	-	-
Athlete 24	National Basketball Association	Small forward	79	225	27	364	Collision with athlete	Lower third	Ankle dislocation	Y

**Figure 2:**
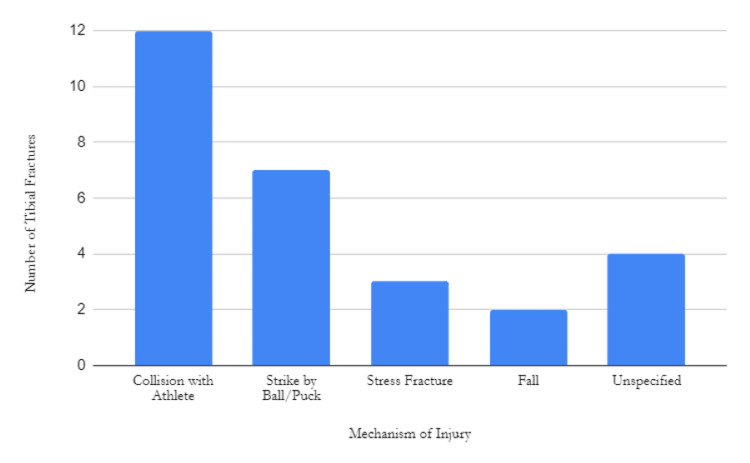
Number of tibial fractures based on mechanism of injury

Ipsilateral lower limb injuries associated with tibial fractures included fibular fractures (21.4%, n=6), MCL and lateral meniscus tear (3.6%, n=1), and ankle dislocations (3.6%, n=1). The mechanisms of injury varied, with 12 fractures resulting from athlete collisions, seven from baseball or hockey puck trauma to the tibia, three from stress fractures, two from athletes tripping or falling, and four unspecified (Figure 2).

**Figure 3:**
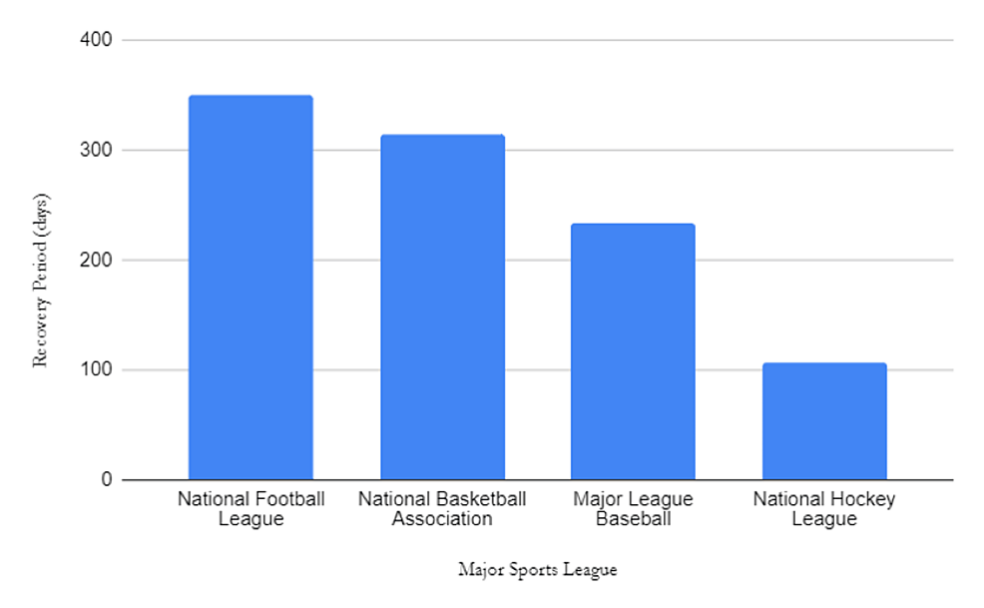
Average time (days) between injury and return to play based on professional sports league

The average age of athletes at the time of injury was 26.96 years. The average time from the date of injury to return to play was approximately 286.75 days. Notably, NFL players experienced the longest recovery time, with an average of 350.18 days before returning to play. NBA athletes followed closely with an average of 314.67 days, MLB players with an average of 234.13 days, and NHL athletes with an average of 106.5 days (Figure 3).

**Figure 4:**
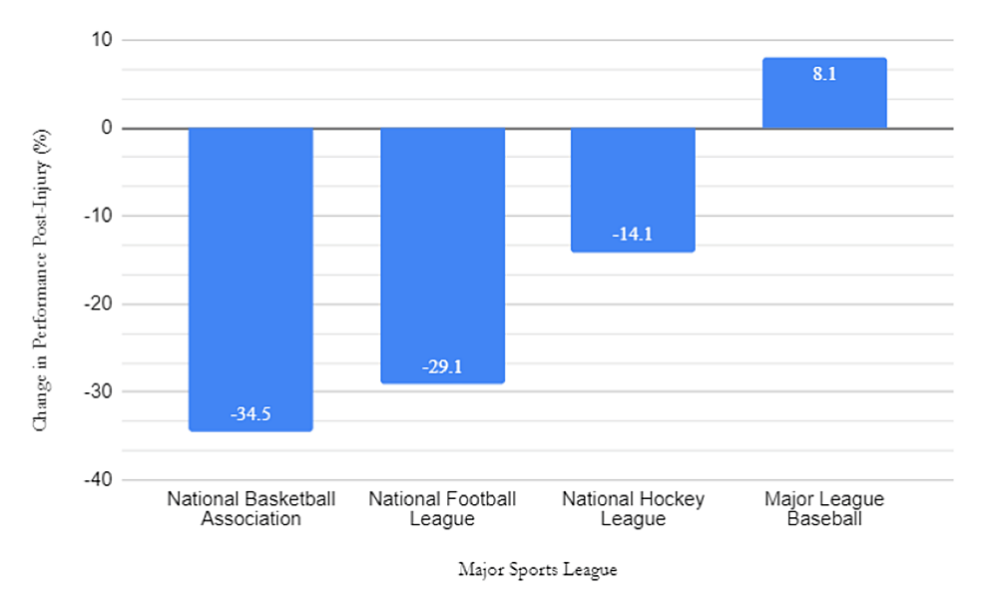
Percent change in athletic performance in the season immediately following tibial fracture versus the season prior to injury

There was no significant correlation between change in athletic performance upon return and athlete weight, height, or age at the time of injury. Upon returning to play, athletes exhibited an overall 14.7% (n=24) decrease in performance comparing the season prior to injury and the season following return across all leagues. Stratifying by league, NBA, NFL, and NHL players experienced performance decreases of 34.5% (n=3), 29.1% (n=10), and 14.2% (n=2), respectively, in the season following their return. Paradoxically, MLB players demonstrated an 8.1% (n=9) increase in performance following their return from tibial fractures (Figure 4).

## Discussion

The majority of tibial fractures occurred in the NFL (n=14) compared to other leagues, which may be attributed to the frequency of collisions intrinsic to the sport. This was consistent with the mechanism of injury analysis as eight of 14 fractures in the NFL were because of athlete collisions. This finding is concordant with trends in the literature [3,8,20,21]. An epidemiologic study in 2020 investigated 18 US sports and recreational activities and subsequently determined that football carried the highest risk of fracture. In their study, football contributed to 22.5% of all the fractures found [22]. However, football may be overrepresented in the aforementioned and present study because matches involved a greater number of players compared to other sports and activities. Furthermore, NFL athletes who were injured had both the longest average time of return (350.18 days) and the second-largest decrease in player performance (-29.1%). The lengthy time to return and performance decrease may be because of the severity of injuries suffered by NFL athletes and the further collisions they are subjected to immediately upon return. Overall, NFL players suffer the most tibial fractures of all professional athletes and have the longest recovery periods with a decrease in performance post-injury. Further study is imperative to guide injury prevention and optimize recovery protocol [3,8,21,23]. 

On average, tibial fracture was associated with decreased athletic performance upon return. Separation by sports leagues revealed a consistent decrease in performance among NFL, NBA, and NHL athletes. However, MLB athletes had a paradoxical increase in performance upon return. While a positive association is likely a result of a small sample size, the vast difference in performance upon return compared to other sports leagues is notable. This result may be because of the isolated nature of injuries suffered by MLB players, with the majority being because of tibial trauma from the direct impact of baseball, sparing other limbs from injury. Other considerations potentially include better rehabilitation programs implemented in the MLB. With the exception of baseball, all sports analyzed were contact sports. This confounding factor could have impacted numerous factors including the number of injuries, time to recovery, and performance after injury. Notably, the NHL had the fewest tibial fractures (n=2) and the shortest period of recovery (106.5 days). Given the high-contact nature of the sport, this finding is unexpected but could be because of the heavy amount of padding that NHL players wear during games. Overall, the results indicate that tibial fractures decrease athletic performance upon returning from injury, with the exception of the MLB. Further investigation needs to be conducted to determine whether this outlier is legitimate, and, if so, why this is the case.

Notably, the NFL and NBA do not carry the same intrinsic risks of tibial fracture from ball or puck strike as the MLB and NHL. The majority of injuries within the NFL and NBA occurred from collisions with an athlete, whereas the majority of injuries in the MLB and NHL were because of ball/puck strikes. With the limited sample size, it is difficult to accurately determine how the mechanism of injury impacts both the length of absence and performance upon return to play, but it is an area of investigation that may yield informative results in the future.

Tibial fractures were not often accompanied by an associated injury. However, when this happened to be the case, the most common associated injury was a fibular fracture. Given their positional anatomy and collision being the most common mechanism of injury, these data are logically consistent. With more data in the future, it would be interesting to see how associated injuries impact time to recovery and performance.

From the sparsely available public data on each athlete’s recovery and interventions for injury, every athlete who suffered a tibial fracture subsequently underwent surgery for management. This finding is unsurprising, but it confirms that surgical intervention for tibial fracture remains the mainstay treatment among athletes [4].

There are several limitations in this study that may impact the validity of the results. Given the specific injury of interest, tibial fracture, our sample size was limited, and we were unable to reach statistical significance in our findings. Additionally, athletic performance is difficult to quantify. Extensive research is continuously ongoing into the statistics that can encapsulate an athlete’s performance, with many experts noting that it is an impossible task because of certain intangible traits that characterize some athletes. While we attempted to quantify athletic performance, many other numerical systems exist that may yield different results [17]. Further investigation can be done on whether these trends are apparent across other performance measures. An additional consideration is that often athletic performance declines with age, which may complicate assessment upon return from injury. Moreover, return-to-play analysis does not account for season start times, team management decisions, and personal obstacles that may impact athlete recovery.

## Conclusions

Tibial fractures, although relatively rare among pro athletes during a season, can have a profound impact on their careers. When athletes experience such fractures, prompt surgical intervention is often necessary, and in unfortunate instances, multiple subsequent procedures may be required. Subsequently, a significant amount of time is devoted to recovery, spanning several months before the athlete can make a comeback. Nevertheless, upon returning to the sport, athletes may notice a decline in their performance. However, further research is needed to establish a stronger correlation between player performance and the recovery process following a tibial fracture.

## Appendices

Appendix 1: Fantasy scoring point values 

NFL Player Performance Numerical Grading System

Passing Yards: 1 point per 25 yards

Passing Touchdowns: 4 points

Passing Interceptions: -2 points

Rushing Yards: 1 point per 10 yards

Rushing Touchdowns: 6 points

Receptions: 1 point (only if using (Point Per Reception) PPR scoring)

Receiving Yards: 1 point per 10 yards

Receiving Touchdowns: 6 points

2-Point Conversions: 2 points

Fumbles Lost: -2 points

Fumble Recovered for a Touchdown: 6 points

Sacks: 1 point

Interceptions: 2 points

Fumbles Recovered: 2 points

Safeties: 2 points

Defensive Touchdowns: 6 points

Kick and Punt Return Touchdowns: 6 points

2-Point Conversion Returns: 2 points

Points Allowed (0): 10 points

Points Allowed (1-6): 7 points

Points Allowed (7-13): 4 points

Points Allowed (14-20): 1 point

Points Allowed (21-27): 0 points

Points Allowed (28-34): -1 point

Points Allowed (35+): -4 points

Solo Tackles: 1 point

Assisted Tackles: ½ point

Sacks: 2 points

Sack Yards: 1 point per 10 yards

Tackles For Loss: 1 point

Quarterback Hits: 1 point

Passes Defended: 1 point

Interceptions: 3 points

Fumbles Forced: 3 points

Fumbles Recovered: 3 points

Defensive Touchdowns: 6 points

2-Point Conversion Returns: 2 points

PAT (Point After Touchdown) Made: 1 point

FG (Field Goal) Made (0-49 yards): 3 points

FG Made (50+ yards): 5 points

MLB Player Performance Numerical Grading System

Singles: 1 point

Doubles: 2 points

Triples: 3 points

Home Runs: 4 points

Runs: 1 point

Runs Batted In: 1 point

Walks: 1 point

Hit By Pitch: 1 point

Stolen Bases: 2 points

Caught Stealing: -1 point

Wins: 4 points

Saves: 2 points

Innings Pitched: 1 point

Earned Runs Allowed: -1 point

NHL Player Performance Numerical Grading System

Goals: 3 points

Assists: 2 points

Shots On Goal: 0.5 points

Plus/Minus: 1 point

Blocks: 0.5 points

Power Play Goals/Assists: 0.5 points

Short-Handed Goals/Assists: 0.5 points

Shootout Goals: 0.2 points

Wins: 3 points

Goals Against: -1 point

Saves: 0.2 points

Shutouts: 2 points

NBA Player Performance Numerical Grading System

Three Point Field Goals: 3 points

Two Point Field Goals: 2 points

Free Throws Made: 1 point

Rebounds: 1.2 points

Assists: 1.5 points

Blocked Shots: 2 points

Steals: 2 points

Turnovers: -1 point
